# Patient experiences of narcolepsy and idiopathic hypersomnia in the Nordics: a patient journey map

**DOI:** 10.1111/jsr.14376

**Published:** 2024-10-26

**Authors:** Märt Vesinurm, Christina Dünweber, Jesper Rimestad, Anne‐Marie Landtblom, Poul Jørgen Jennum

**Affiliations:** ^1^ Nordic Healthcare Group Oy Helsinki Finland; ^2^ Department of Industrial Engineering and Management Aalto University School of Science Espoo Finland; ^3^ Takeda AS Vallensbæk Strand Denmark; ^4^ Takeda AS Asker Norway; ^5^ Department of Medical Sciences, Neurology Uppsala University Uppsala Sweden; ^6^ Department of Biomedical and Clinical Sciences Linköping University Linköping Sweden; ^7^ Department of Clinical Neurophysiology, Rigshospitalet Danish Center for Sleep Medicine Copenhagen Denmark

**Keywords:** central disorders of hypersomnolence, idiopathic hypersomnia, lived experience, living with narcolepsy, patient experience, patient journey

## Abstract

Central disorders of hypersomnolence (CDH) are chronic diseases that significantly impact the lives of affected individuals. We aimed to explore the perspectives of individuals with narcolepsy type 1 (NT1), narcolepsy type 2 (NT2), and idiopathic hypersomnia (IH), and the challenges they encounter in their daily lives and within the healthcare systems in the Nordics. Interviews with patients (*N* = 41) and healthcare professionals (*n* = 14) and a patient survey (*n* = 70) were conducted in 2022 in Denmark, Sweden, Finland, and Norway to develop a patient journey map that visualises the patient with CDH journey and provides insights into the difficulties faced by these individuals. The patient journey mapping approach was chosen to focus on the processes and experiences of patients, highlighting the challenges they confront. Our findings revealed that the process of receiving a CDH diagnosis, as well as subsequent misdiagnoses and treatment, can be protracted and burdensome. CDH diagnoses remain poorly understood by neurologists, general practitioners, and the public, resulting in adverse consequences, with patients reporting a mean (standard deviation [SD]) time from symptom onset to diagnosis of 8.4 (5.11) years and a mean (SD) of 5.5 (4.17) productive hours lost/day. The available non‐pharmaceutical support for patients with CDH, encompassing medical, psychological, educational, and professional assistance, was insufficient. The generalisability of the findings to one specific diagnosis is limited due to the collective analysis of the CDH. These findings are invaluable for identifying disruptions in the patient with CDH journeys and for designing improved pathways for those with NT1, NT2, and IH in the future.

## 1 INTRODUCTION

Central disorders of hypersomnolence (CDH), such as narcolepsy type 1 (NT1), narcolepsy type 2 (NT2), and idiopathic hypersomnia (IH), are characterised by excessive daytime sleepiness (EDS). In this study, NT1, NT2, and IH are collectively referred to as CDH for the reader's convenience. CDH are rare diseases, with narcolepsy having an estimated incidence of 0.74 per 100,000 person‐years and a prevalence of around 25–50 per 100,000 people and typical onset in early adulthood (Acquavella et al., 2020; Kallweit et al., 2022; Kornum et al., 2017; Longstreth et al., 2007). There are two major types of narcolepsy—NT1 and NT2—the former distinguished by symptoms of cataplexy and a loss of orexin‐producing neurones in the lateral hypothalamus. Low to absent orexin levels in the cerebrospinal fluid support a diagnosis of NT1. IH is another type of CDH, characterised by EDS, no sleep‐onset rapid eye movement (REM) period, and associated with long sleep duration and severe sleep inertia (Billiard & Sonka, 2016). EDS is a common feature among all these disorders, but each has different underlying causes, symptomatology, and severity (Lammers et al., 2020).

Central disorders of hypersomnolence have a significant negative impact on various aspects of life, including health, social interactions, education, and work‐related activities for both individuals living with the disease and their families (Dodel et al., 2007; Jennum et al., 2017; Jennum et al., 2020; Raggi et al., 2019). They are associated with high rates of under‐diagnosis, late diagnosis, and misdiagnosis, which contribute to the social and comorbid prognosis that patients with narcolepsy face (Jennum et al., 2012; Jennum et al., 2013; Kornum et al., 2017).

Once a correct diagnosis is established, there are several pharmacological and non‐pharmacological treatment options available (Bassetti et al., 2021; Lammers et al., 2020). However, there are still significant gaps in understanding the burden that patients with CDH experience. Following the increase in incidence of NT1 after the H1N1 influenza pandemic and the Pandemrix vaccination, there has been increased awareness and research into narcolepsy and its causal mechanisms, genetic as well as environmental (Gauffin et al., 2024; Hallberg et al., 2019; Melen et al., 2013; Sarkanen et al., 2018; Verstraeten et al., 2016). Research has also focused on the cost‐effectiveness of different treatment options (e.g., Bolin et al., 2017; Bolin et al., 2020; Gauffin et al., 2022), the socioeconomic cost of narcolepsy (e.g., Jennum et al., 2020), and the development of screening and quality‐of‐life tools (Bargiotas et al., 2019; Dauvilliers et al., 2017; Hublin et al., 1994). However, there is a need for more research from the patients’ perspectives to understand how individuals with CDH experience their patient journey and how it deviates from the ideal pathway (Vesinurm et al., 2024).

Health‐related quality of life (HRQoL) is significantly reduced in patients with CDH. These patients require assistance with medication renewal and psychological, educational, and social support (Dodel et al., 2007; Raggi et al., 2019). Early diagnosis and improved treatment could potentially reduce the disease burden and have a significant socioeconomic impact, as well as improve HRQoL for patients (Ervik et al., 2006; Jennum et al., 2009).

Here, we present the patient with CDH journey map from the perspective of NT1, NT2, and IH, exploring how patients experience their disease in the Nordics. We visualise the patient journey and discuss the challenges faced at different stages, from the onset of symptoms to the management of daily life with CDH. By examining these conditions collectively, we sought to acknowledge the ambiguity that can be present in the diagnostic process for the patients and understand the shared experiences and challenges faced by patients with CDH as they navigate the diagnostic and treatment processes, and life after the diagnosis.

## 2 METHODS

The methodological approach is patient journey mapping (PJM), which is a relatively novel method for collecting and organising insights on how specific patient groups experience their patient journeys and the challenges they face. This approach focuses on understanding the process through which patients navigate the healthcare system and identifying the various touchpoints between stakeholders (Davies et al., 2023; Madathil et al., 2020; McCarthy et al., 2016; Trebble et al., 2010). In addition, combining the experiences of patients with the perspectives of healthcare professionals (HCPs) has been shown to elicit further insights into the care process (Larsen et al., 2024).

**FIGURE 1:**
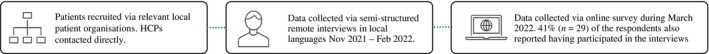
Visualisation of the data collection process.

The data were collected between November 2021 and February 2022. The data collection process involved semi‐structured interviews with HCPs (*n* = 14) and patients living with a self‐reported diagnosis of CDH (NT1, NT2, or IH) (*n* = 41), as well as a subsequent online patient survey (*n* = 70). The interviews and the survey were conducted by experienced service designers and consultants of Nordic Healthcare Group, who were fluent in the local languages of Finland, Denmark, Sweden, and Norway. All interviewers had extensive experience in conducting interviews. The data collection process is depicted in Figure 1. Based on the field notes from the semi‐structured interviews and the survey results, a PJM was developed, which can be found in its entirety in Appendix A.

The inclusion criteria specified that the participant was aged >18 years and be diagnosed (self‐reported) with NT1, NT2, or IH. All patient interviewees were informed about the opportunity to participate in the interview through an information kit developed in collaboration with local patient organisations. This kit was shared on closed social media channels of the patient organisations, where volunteers could find a link to a consent form and provide their contact information. All contact information was deleted after analyses were performed. Before each interview, the interviewees were asked to confirm their consent to participate. All participants gave their informed consent.

**FIGURE 2:**
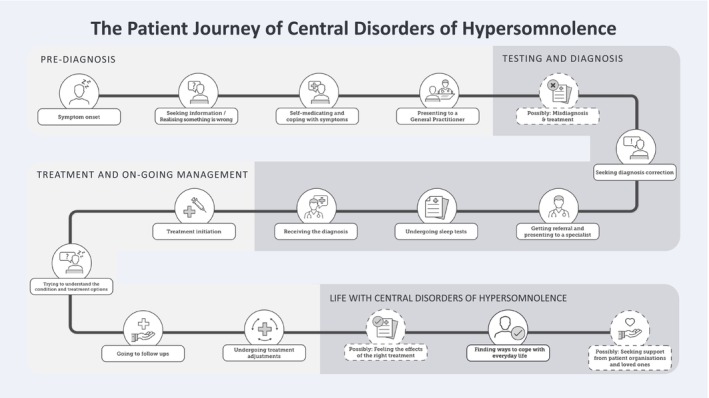
Structure of the patient journey map of central disorders of hypersomnolence.

The interviewers underwent training on CDH, which included a primer on the disease and conducting extensive desktop research on common discussion topics related to CDH. They familiarised themselves with information provided by local patient organisations and the latest publications on CDH. The interviewers also carefully reviewed the interview guides (Appendix B and Appendix C), which were translated into the local languages. The raw dataset collected consisted of the field notes taken during these interviews. The field notes taken by the interviewers were then transferred into an Excel spreadsheet and insights were categorised to follow the basic structure of the patients’ journey from pre‐diagnosis through getting the diagnosis to living with the disease as laid out in Figure 2. Subsequently, the interviewers summarised each interview using a web‐based workshop tool. These summaries along with the Excel spreadsheet, were then analysed using qualitative content analysis (Graneheim & Lundman, 2004). An example of the coding of interview data is provided in Table 1. By combining insights from different countries, a joint Nordic PJM was elicited. This map was further enhanced with country‐specific details whenever there were notable deviations from the other countries’ results. The final PJM is presented in Appendix A.

**TABLE 1:** Examples of coding interview data.

First order dimension	Second order dimension	PJM dimension
‘Narcolepsy patients do not have nightmares, they live them.’ (Interviewee 2, Finnish, NT1)	CDH can be very scary, especially at symptom onset.	Pre‐diagnosis
‘The diagnosis was partly a huge relief because then I was not mentally ill as some doctors thought during my second childbirth. But also scary, I could never be able to rest properly again, where should I solve life with two very young children?’ (Interviewee 31, Swedish, NT1)		
‘I could be worried that the disease could suddenly worsen or there could be long‐term side effects of medicines not known today. I could also be worried if I lose my job, I can no longer afford the most expensive medication and have to take a cheaper one which does not help.’ (Interviewee 28, Swedish, NT1)		
‘I saw five to six doctors, but none of them took me seriously, saying that there is nothing wrong with me, we are all tired.’ (Interviewee 3, Finnish, NT1)	There is a lack of knowledge among HCPs about CDH and it can be difficult for patients to be taken seriously	
‘I was thought to be an attention‐seeking teen with joint problems.’ (Interviewee 7, Finnish, NT2)		
‘We have taken it upon us to teach both medicine students and other health care professionals about this disease ‐ even though it is very rare. We hope that this can help us catch people earlier on so that they can get the help they need and hopefully live a life more similar to that of their peers.’ (Interviewee 54, HCP, Neurologist)		

Abbreviations: CDH, central disorders of hypersomnolence; HCP, healthcare professional; NT1, narcolepsy type 1; NT2, narcolepsy type; PJM, patient journey map.

*Note*: Interview notes were analysed through three dimensions.

## 3 RESULTS

### 3.1 Informant characteristics

Data from the interviews were collected from patients with NT1 (*n* = 30), NT2 (*n* = five to seven), and IH (*n* = four to six). The analyses were not separated by the three diagnoses but under the basic assumption that the patient journey of these CDH would be largely similar. The interviews with patients (*n* = 41) and HCPs (*n* = 14) lasted between 60 and 90 min. Among the HCPs, nine were neurologists, and the remaining five were other relevant HCPs. All interviewed HCPs had experience with CDH and regularly interacted with patients with CDH in their current positions. Convenience sampling was used to select the HCPs from the pool of Nordic professionals working with patients with CDH and they were compensated for their contribution. All interviews were conducted using Microsoft Teams.

The interviewed patients, of whom 90% were female, ranged in age from 20 to 57 years. The distribution of patient interviews across the studied countries was as follows: 10 from Denmark, 10 from Finland, 11 from Norway, and 10 from Sweden. Among the patients interviewed, 30 reported having NT1, five reported having NT2, and four reported having IH. Two of the Danish interviewees were unsure whether they had NT2 or IH. A summary of the participants’ data can be found in Table 2.

**TABLE 2:** For the creation of the patient journey map, 41 patients and 14 healthcare professionals were interviewed from four different Nordic countries.

Variable	Denmark	Finland	Norway	Sweden	Nordic
Age, years, mean, median	39.2, 38.5	34.3, 28.5	33.2, 30	37.6, 37.5	36, 32
Female, *n/N* (%)	9/10 (90)	10/10 (100)	9/11 (81.8)	9/10 (90)	37/41 (90)
NT1, *n/N* (%)	6/41 (14.6)	9/41 (22.0)	6/41 (14.6)	9/41 (22.0)	30/41 (73.2)
NT2, *n/N* (%)	2/41–4/41^a^ (4.9–9.8)	1/41, 2.4	2/41 (4.9)	0/41 (0)	5/41–7/41^a^
IH, *n/N* (%)	0/41–2/41^a^ (0–4.9)	0/41, 0	3/41 (7.3)	1/41 (2.4)	4/41–6/41^a^
Total, *n/N* (%)	10/41 (24.4)	10/41 (24.4)	11/41 (26.8)	10/41 (24.4)	41/41 (100)
**HCPs, *n/N* (%)**					
Neurologists	2/14 (14.3)	2/14 (14.3)	3/14 (21.4)	2/14 (14.3)	9/14 (64.3)
Other	1/14 (7.1)	1/14 (7.1)	1/14 (7.1)	2/14 (14.3)	5/14 (35.7)
Total	3/14 (21.4)	3/14 (21.4)	4/14 (28.6)	4/14 (28.6)	14/14 (100)

Abbreviations: HCP, healthcare professional; IH, idiopathic hypersomnia; NT1, narcolepsy type 1; NT2, narcolepsy type.

^a^ Two of the Danish interviewees were unsure whether they had NT2 or IH.

An online survey was conducted in March 2022 to gather additional data on the main topics identified during the patient interviews. The survey was sent out to 165 patients who had initially expressed interest in participating in the interviews. Out of these, 70 completed the survey (42% response rate), among whom 29 had also participated in the interviews. A summary of the survey respondents can be found in Table 3. The structure of the survey is provided in Appendix D.

**TABLE 3:** The interview data were further enriched with an online survey (*N* = 70).

Patients, *n/N* (%)	Denmark	Finland	Norway	Sweden	Nordic
NT1	13/70 (18.6)	18/70 (25.7)	10/70 (14.3)	9/70 (12.9)	50/70 (71.4)
NT2	4/70 (5.7)	2/70 (2.9)	1/70 (1.4)	0/70 (0)	7/70 (10)
IH	0/70 (0)	0/70 (0)	13/70 (18.6)	0/70 (0)	13/70 (18.6)
Total	17/70 (24.3)	20/70 (28.6)	24/70 (34.3)	9/70 (12.9)	70/70 (100)

Abbreviations: IH, idiopathic hypersomnia; NT1, narcolepsy type 1; NT2, narcolepsy type.

### 3.2 Patient journey map: pre‐diagnosis

#### 3.2.1 Seeking help for CDH symptoms

Interviewees reported noticing an increased need for sleep, usually followed by confusion about the sudden and continuous feeling of exhaustion, not suspecting anything serious until episodes of cataplexy (if any) occurred. Typical early symptoms included: EDS, cataplexy (for some), fragmented night‐time sleep (sometimes including sleep paralysis, hallucinations, and parasomnias), and brain fog. These symptoms caused difficulties at work or school, feelings of exhaustion, or even falling asleep.

‘Narcolepsy patients do not have nightmares, they live them.’ (Interviewee 2, Finnish, NT1)

Some interviewees reported starting to look for answers online once they realised something was wrong. They searched for information, some stumbling across the possible diagnosis, but having difficulty explaining what they were experiencing. Interviewees reported resorting to self‐medication with caffeine, energy drinks, sugar, or alcohol in an attempt to cope with their symptoms. They frequently sought help from school or occupational healthcare, with the result of only being sent home to rest, which led the patients to search the internet for explanations for their symptoms. Help was sought many times, leaving many feeling that they were not taken seriously. It may take years to finally get a referral to a neurologist or a sleep clinic.

‘I saw five to six doctors, but none of them took me seriously, saying that there is nothing wrong with me, we are all tired.’ (Interviewee 3, Finnish, NT1)

‘I was thought to be an attention‐seeking teen with joint problems.’ (Interviewee 7, Finnish, NT2)

Only 3% (two of 70) of the survey respondents reported being correctly diagnosed by their general practitioner (GP). Most patients needed to present the suspicion of CDH and convince their GPs to give the referral to neurology. This challenge was also recognised by the HCPs: ‘We have taken it upon us to teach both medicine students and other HCPs about this disease – even though it is very rare. We hope that this can help us catch people earlier on so that they can get the help they need and hopefully live a life more similar to that of their peers.’ (Interviewee 54, Danish HCP, Neurologist).

### 3.3 Patient journey map: testing and diagnosis

#### 3.3.1 Being misdiagnosed

Misdiagnoses at the beginning of the patient journey were reported to have significant consequences for individuals with CDH. Examples include receiving the wrong medication, facing difficulties in studies, acquiring, or keeping a job, and challenges in developing relationships. Interviewees expressed frustration and worry as they had visited multiple doctors and received different diagnoses before finally obtaining the correct one.

‘After a few years we (twins) went to our GP, who suggested that the exhaustion was due to being overweight and so we both got a gastric bypass. This did not help.’ (Interviewee 40, Danish, NT1)

The HCPs acknowledged the challenges faced by GPs in diagnosing CDH, as the symptoms can mimic other conditions. Limited education and awareness of the conditions often result in ruling out various diagnoses before arriving at the correct one. Specialists interviewed also recognised the harm caused by treating patients with medication intended for a different disease, as it may mask key symptoms of narcolepsy and further delay the correct diagnosis.

‘The problem is that to get a referral to a specialist, the patient first has to address that something isn't right and then be able to tell a doctor about the symptoms, a doctor who might not have the knowledge to suspect narcolepsy.’ (Interviewee 42, Finnish HCP, Other)

Of the survey respondents, 27% (*N* = 19) reported initially being diagnosed with something other than their current diagnosis based on their symptoms. The reported initial diagnoses included bipolar disorder, insomnia, hypothyroidism, depression, burnout, exhaustion, epilepsy, sleep apnea, anxiety, restless legs, and attention deficit hyperactivity disorder. This highlights the wide range of different diagnoses that patients with narcolepsy encounter.

#### 3.3.2 Getting a CDH diagnosis

Due to many patients with CDH not being taken seriously and/or being initially misdiagnosed, the time from symptom onset to the right diagnosis can be very long. By the time patients are finally referred for further evaluation, they already face significant difficulties in their daily lives, struggling to manage school or work, pursue hobbies, or maintain a social life. As described by one of the interviewees: ‘The doctors believed that if I was able to study and work full time, I could not have any serious illnesses. HCPs told me I was healthy and therefore I doubted myself for almost 10 years and tried to behave and pretend like I was doing well.’ (Interviewee 13, Norwegian, IH).

The HCPs emphasised that the issue lays not only in misdiagnosis but also in the lack of diagnosis altogether, as the process of obtaining the correct diagnosis is often lengthy. As one HCP put it ‘Many wrong tests are done before a lumbar punction will show NT1 or NT2 and it can take 5–10 years to get a diagnosis.’ (Interviewee 50, Swedish HCP, Neurologist).

The mean (standard deviation [SD]) time from symptom onset to diagnosis reported by patients in the survey was 8.4 (5.1) years. The delay in diagnosis varied significantly among the studied countries, ranging from a mean (SD) of 4.6 (3.7) years in Sweden to 12.4 (6) years in Denmark. It also varied among different subtypes of narcolepsy, with a range of a mean (SD) of 7.3 (5.1) years for NT1 to 11.7 (5.0) years for NT2 and 10.8 (5.4) years for IH. These findings highlight the challenges of delayed diagnosis faced by patients with CDH, with 20% of survey respondents ranking diagnosis delay as their top challenge (Table 4).

**TABLE 4:** The patient‐reported time from symptom onset to diagnosis varied greatly between the four Nordic countries.

Variation by diagnosis	Time from symptom onset to diagnosis, years, mean (SD)	Number of patients
NT1	7.3 (5.1)	50
NT2	11.7 (5.0)	7
IH	10.8 (5.4)	13
Total	8.4 (5.1)	70
**Variation by region**		
Finland	6.4 (3.7)	20
Sweden	4.6 (3.7)	9
Norway	8.6 (4.8)	24
Denmark	12.4 (6.0)	17
Total	8.4 (5.1)	70

Abbreviations: IH, idiopathic hypersomnia; NT1, narcolepsy type 1; NT2, narcolepsy type; SD, standard deviation. *Source*: CDH online survey.

### 3.4 Patient journey map: treatment and on‐going management

#### 3.4.1 Finding suitable medication and treatment

Interviewees reported that finding the right medication could be a burdensome process of trial and error trying to find a balance between symptom management and dealing with side effects. Despite trying all available options, many still struggled to find a suitable treatment. In all, 33% (23 of 70) of the survey respondents reported no improvement in HRQoL from treatment and those that reported an improvement, experienced a mean (SD) lag of 1.6 (2.8) years from the beginning of treatment to an experienced improvement.

‘What is quality of life, – finding a new philosophy for life? Life should be about being able to contribute, to be able to create, but love also fills a lot and all the support I have got from my husband. Everything is strenuous today and quality of life has become so much worse only love counteracts it.’ (Interviewee 24, Swedish, NT1)

The HCPs agreed that while patients were treated for their symptoms, the underlying disease remains unresolved. Episodes, such as nightmares and fatigue pose significant challenges in terms of treatment, and predicting the overall effect of medication on patients is difficult. Both the patients and HCPs also called for more non‐pharmacological treatment approaches:

‘It is important to talk about more than just medication to treat tiredness. The patient needs routines, exercise, a place of work/school to make it clear that there is a beginning and an end of a day.’ (Interviewee 50, Swedish HCP, Neurologist).

‘Treatment should focus more on non‐pharmacological treatment, mental health and include a multidisciplinary team of experts such as nutritionists, physical therapy and psychiatric help.’ (Interviewee 10, Finnish, NT1)

#### 3.4.2 Follow‐ups with specialists

Interviewees reported that follow‐ups with specialists did not provide much value and some expressed that they had too limited contact with specialists, especially in the later stages of their patient journey due to having their follow‐up care administered by a GP, who is not an expert in their condition.

‘I do not at all experience any value of our yearly meeting – it most of all seems like a meeting that just needs to be done because some kind of protocol mentions it.’ (Interviewee 39, Danish, IH)

‘I feel completely alone regarding my diagnosis – my GP is not an expert… I am quite sad about that.’ (Interviewee 38, Danish, NT1)

The interviewees expressed dissatisfaction with the main focus of specialist follow‐ups, which was primarily on medication and prescription renewals. The frequency of follow‐ups varied greatly, ranging from every 3 months to once a year. Some patients felt that most of the time during these follow‐ups was spent on completing questionnaires about the effectiveness of the treatment.

The HCPs commented that due to lack of knowledge, many doctors feel more comfortable focusing on medication. They also mentioned that patients often take insufficient notes, fail to track their symptoms adequately, and struggle to recall events since their previous follow‐up. This lack of comprehensive information makes it challenging for doctors to have a holistic view of the patient's condition. Additionally, the absence of standardised care for narcolepsy and IH and the limited knowledge among neurologists about the conditions further hinder the comprehensiveness of patient care.

The lack of keeping track of their condition was also reflected in the survey responses, where 73% (51 out of 70) respondents answered ‘I do not actively keep track of my condition’ when asked how they keep track of their condition. In all, 19% reported using digital tools to track their sleep and 11% reported using a paper journal.

#### 3.4.3 Lack of psychological support

The interviewees expressed a need for psychological support in dealing with the challenges of their lives but felt that their specialists primarily focused on medicinal aspects and did not provide adequate non‐pharmacological support. Some patients shared their disappointment in seeking help from psychologists who did not fully understand the experience of living with narcolepsy: ‘It was so depressing to realise that after doing so much myself to visit a psychologist, the visits provided me no value because the psychologist didn't understand what it's like to live with narcolepsy.’ (Interviewee 5, Finnish, NT1).

The HCPs acknowledged the importance of psychological care and rehabilitation for patients. They believed that it should be easy for patients to receive referrals for such support, given the short duration of doctors’ visits. Some pinpointed the lack of resources as the problem.

‘They need more accessible medicine, there are no narcolepsy teams, other than at Karolinska, to ensure the quality of life for these patients. The patients are very alone in the system and there is a gap where the patient (from all aspects, mentally, physically, and spiritually) is not, and cannot, be treated well today, because there aren't enough resources.’ (Interviewee 50, Swedish HCP, Neurologist)

‘Narcolepsy teams including different professions are needed to support the patient.’ (Interviewee 49, Swedish HCP, Neurologist)

### 3.5 Patient journey map: life with the diagnosis

#### 3.5.1 Coping with daily life

Interviewees reported a lack of information regarding coping strategies or methods to maintain a relatively ‘normal’ life with their condition. This burden was particularly felt by those who already struggled with limited time and energy to care for themselves and their loved ones.

‘Like learning to live a whole new life, new language and new culture, like being an Alien.’ (Interviewee 23, Swedish, NT1)

The HCPs acknowledged this lack of support for newly diagnosed patients. After diagnosis, patients are left to find information on their own, and the limited knowledge among HCPs, especially GPs, leaves patients feeling lonely and forgotten. Information about CDH is often provided verbally and contact information for patient organisations is given.

‘The patient needs practical information in the beginning, to help guide them into a functional life with narcolepsy.’ (Interviewee 44, Finnish HCP, Neurologist)

Interviewees also mentioned the crucial role of patient organisations in providing peer support. On the other hand, some did not wish to identify as ‘a narcolepsy patient’ and would rather distance themselves, referring to the tone of such groups as ‘rather negative’.

‘If there could have been a decisive moment in the journey of illness then it would have been to be able to meet other patients earlier. It would be good for patient organisations to help creating a support network of like‐minded people – it would be good to be able to network with patients in the same district or region, it would have made a big difference for me, because loneliness was just devastating.’ (Interviewee 19, Norwegian, IH).

‘I do not want to be labelled as a narcolepsy patient.’ (Interviewee 7, Finnish, NT2)

#### 3.5.2 General awareness about CDHs and social stigma

Interviewees expressed a burden when it came to explaining their diagnosis to others, as they felt there was a need to ensure that colleagues, friends, and acquaintances were aware of their medical condition. However, they also faced the challenge of dealing with stigma and misinformation. Several instances of being teased in school, labelled as lazy in university, or receiving misguided advice from others on how to stay awake were reported.

‘The hardest part has been that people do not understand the disease, not even one's wise colleagues… Many colleagues were just upset about when I fell asleep in the middle of classes and in the middle of meetings and said I was a bad role model for youth. They thought I could do some exercise, and it would magically help somehow.’ (Interviewee 18, Norwegian, NT1)

Discussing their disease or condition with friends, family, and colleagues, the interviewees reported often encountering a narrow perception that associates the conditions solely with sleeping problems. People fail to grasp the multidimensional challenges of living with narcolepsy, assuming that individuals cannot work if they are constantly falling asleep.

‘I think my mom denies it totally because she does not want to accept that she has been mistaken my whole life about me not being tired due to laziness.’ (Interviewee 36, Danish, NT2)

#### 3.5.3 Lack of facilitation at work or school to better cope with CDH

**FIGURE 3:**
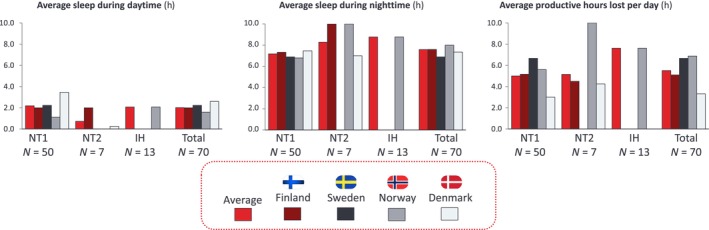
Self‐reported average sleep during daytime, average sleep during night‐time, and average productive hours lost per day due to condition (Source, CDH online survey, *N* = 70, more detailed numbers provided in Table 2). IH, idiopathic hypersomnia; NT1, narcolepsy type 1; NT2, narcolepsy type 2.

Interviewees reported a multifaceted lack of accommodations to better cope with their condition at work or school. Some reported looking for unconventional places like bathrooms and closets to take naps in, just to cope with the symptoms of daytime exhaustion while at work or school. Some even changed jobs, while others found it challenging to reach an understanding with their employers regarding necessary accommodations for someone with CDH. Symptoms such as EDS are frequently misunderstood by teachers, colleagues, and managers, who may mistakenly interpret them as laziness or mere tiredness. Additionally, survey respondents reported a mean (SD) of 5.5 (4.17) h productive hours lost during the day due to their condition, and a mean (SD) of 2 (2.98) h of sleep during the daytime (Figure 3).

## 4 DISCUSSION

To the best of our knowledge, this publication represents the first comprehensive exploration of the patient experiences of CDH expressed as a patient journey. Previous studies have primarily examined quality‐of‐life measures, morbidity, and the educational and welfare consequences of these conditions (Dodel et al., 2007; Ervik et al., 2006; Goswami, 2010; Jennum et al., 2013; Jennum et al., 2017). In this PJM, we sought to delve into the patient's viewpoint and complement it with insights from HCPs. By incorporating both patient and HCP perspectives, we offer a comprehensive understanding of the challenges encountered by patients with CDH throughout their journey. It sheds light on the need for improved education and awareness among HCPs and the general public to better support individuals living with CDH.

The findings of this PJM reveal a wide range of limitations experienced by patients throughout their journey. It is important to note that the challenges faced by the patients extend beyond the symptoms of the condition itself. While the interviews with HCPs provided valuable insights, there were notable differences in the perspectives raised by the HCPs compared to those expressed by the patients, such as whereas the patient interviewees experienced a lack of value from follow‐up visits and an excessive focus on pharmacological therapies, the HCPs noted a lack of self‐monitoring (i.e., with a journal) by the patients as one of the causes of this focus.

### 4.1 Challenges

This PJM highlights several challenges faced by patients that warrant attention and further investigation. One key area of concern is the diagnostic process, which patients often find difficult, slow, and burdensome. Delayed diagnosis and misdiagnosing in CDH are well known, with previous studies having also documented a lack of awareness of narcolepsy in the form of underdiagnosis, late diagnosis, and lack of focus/support in teaching, social, and educational contexts (e.g., Feldman, 2003; Taddei et al., 2016). Of particular interest are the patients’ experiences with clinical management, as they frequently expressed dissatisfaction with the overall management of their condition. It is notable that although it is known from previous literature that patients with CDH frequently encounter cognitive challenges, confirmed by radiological biomarkers (Engström et al., 2014; Witt et al., 2018), these challenges were not brought up by the interviewees.

Many reported a lack of support in managing these challenges, both psychologically and cognitively. Furthermore, patients often face difficulties in having their condition recognised and understood by various environments such as schools, authorities, and workplaces. There is a clear lack of information and knowledge about the disease in these settings. It is crucial for educational institutions, businesses, social agencies, and the healthcare system to consider these factors when dealing with people living with CDH.

While the healthcare system tends to prioritise pharmacological treatment and primary symptoms such as EDS and cataplexy, there is a noticeable lack of attention given to non‐pharmacological support. It is striking that there is a wealth of systematic knowledge regarding pharmacological treatment, but very little regarding support measures, educational and vocational programmes, and interventions in these areas. To better meet the expectations of patients, more attention must be directed towards addressing their specific needs.

### 4.2 Limitations and future research

While the PJM provides valuable insights into the experiences of patients with CDH from various perspectives, it is important to acknowledge the limitations of the PJM. Firstly, the recruitment of patient interviews and survey respondents relied solely on patient organisations and HCPs using convenience sampling, which may introduce selection bias into the data. Similarly, participation bias may influence the findings, as participants may have different characteristics or experiences compared to those who chose not to participate (i.e., more severe symptoms, more proactive attitudes towards their condition, or more positive or negative experiences with the healthcare system). The study was also conducted during the COVID‐19 pandemic, which may have influenced the results due to possible short‐term challenges in accessing healthcare, effects of the pandemic on the mental health of the respondents, or other pandemic related biases that may have affected the experiences of the respondents.

Second, the selection of informants was based on a self‐reported diagnosis, not confirmed by a clinician. In addition, most of the interviewed patients had received a late diagnosis in their patient journey, which could introduce bias when compared to patients who were diagnosed earlier. Furthermore, no data were analysed on different comorbidities of the respondents, which might have contributed to the loss of productive hours per day or hours of sleep during the night, and the overall patient journey. Similarly, the study did not consider quantitative measures such as the Epworth Sleepiness Scale, the frequency of cataplectic attacks, or HRQoL measures.

Third, the overall number of informants (~10 per country) is relatively low, and the gender distribution was 90% female. Although this may limit the generalisability of the findings, it is worth noting that CDH are very rare and a total of 55 interviews and a follow‐up survey with 70 individuals is more than standard for a qualitative inquiry.

Fourth, there are variations in the patient samples across the four countries, and not all issues presented in the study may fully reflect the treatment situations in each country and the specific differences between the countries were not in focus for this study. Some countries may have addressed certain challenges better than others. Similarly, the study did not investigate differences between different geographical regions within the countries (i.e., urban and rural areas).

Fifth, while the data were collected from patients with NT1, NT2, and IH, the content analysis of interviews did not separate the groups, as no significant differences were anticipated between their patient journeys. There may be some differences between the patient journeys, such as the possible earlier diagnosis of NT1 due to a the very distinctive cataplexy symptom. While each condition has distinct characteristics, the focus of this study was on understanding the collective experiences and challenges faced by patients with CDH. This broader perspective allows for identifying common issues and areas for improvement across the spectrum of CDH, rather than isolating each condition. We acknowledge the inclusion of patients with unclear diagnoses (NT2 versus IH) as a limitation, but believe that excluding these patients would reduce the representativeness of our sample and overlooked important insights into the diagnostic challenges faced by this subgroup as, in fact, it is quite an interesting finding that someone living with as serious a disease as NT2/IH would be unsure of their diagnosis, which could either stem from the diagnostic process, which focuses in on the correct diagnosis as more information becomes available or poor awareness of their own condition. The administered survey may be criticised for not utilising standardised instruments and instead delving deeper into topics already brought up during the interviews. Lastly, the study did not include children and their parents, highlighting the need for further exploration of these issues.

Overall, the insights from the PJM reveal a significant unmet need for further investigation into these limitations and areas of concern. Future studies should further investigate the specific differences between the patient journeys of NT1, NT2, and IH beyond that of possible earlier diagnosis. In addition to the separate diagnoses, specific differences in the care processes and patient journeys between different countries and regions within countries should be investigated. Furthermore, the difference between patient journeys considering different comorbidities, HRQoL, and symptom severity should be considered.

## 5 CONCLUSIONS

By utilising PJMs, we can effectively analyse the patient's trajectory within the healthcare system, enabling HCPs to identify crucial points of interaction between patients and healthcare providers. This understanding provides valuable insights into the functionality of the healthcare system and opportunities for improvement. By comprehending the patient's experience, HCPs can develop educational materials and protocols to enhance staff training, resulting in improved patient care. Furthermore, by recognising the societal impact of the patient journey, healthcare providers can devise strategies to reduce costs and enhance access to care for all individuals. HCPs can gain valuable insights into enhancing healthcare practices, including improved diagnostic procedures and reduced misdiagnosis, particularly in complex patient journeys such as CDH. These findings can serve as a foundation for developing standardised methods to measure and manage patients with CDH journeys, while also quantitatively evaluating the extent of the challenges faced.

## AUTHOR CONTRIBUTIONS

**Märt Vesinurm:** Conceptualization; methodology; writing – original draft; writing – review and editing; formal analysis. **Christina Dünweber:** Conceptualization; project administration; resources; writing – review and editing. **Jesper Rimestad:** Writing – review and editing; validation. **Anne‐Marie Landtblom:** Writing – review and editing; validation; supervision. **Poul Jørgen Jennum:** Writing – review and editing; supervision; validation.

## FUNDING INFORMATION

The project and this work were funded and initiated by Takeda Pharma AB, a subsidiary of Takeda Pharmaceuticals Inc.

## CONFLICT OF INTEREST STATEMENT

The authors declare the following conflicts of interest: Märt Vesinurm is employed by Nordic Healthcare Group Oy, Helsinki, Finland. Christina Dünweber is employed at Takeda Pharma Denmark A/S. Jesper Rimestad is employed at Takeda Pharma Norway A/S. Dr Anne‐Marie Landtblom reports personal fees from Takeda AB, Jazz Pharmaceuticals, and UCB, as well as a research grant from Aoporphan drugs. Dr Poul Jørgen Jennum reports personal fees from Takeda and an advisory board, Takeda 2021. Both Dr Anne‐Marie Landtblom and Dr Poul Jørgen Jennum disclosed reimbursement for their contributions to the interviews during the work on the Nordic narcolepsy patient journey mapping.

## Data Availability

The data that support the findings of this patient journey mapping are available on reasonable request from the corresponding author (M.V.). The data are not publicly available due to restrictions in relation to information that could compromise the privacy of interviewees. The data will be provided after its de‐identification, in compliance with applicable privacy laws, data protection, and requirements for consent and anonymisation.

## APPENDIX B Narcolepsy PATIENT interview guide.

### B.1

Information for the interviewer:

The following document describes the interview process and contains an overview of the interview questions.

The interviews will take ~70–90 min. Take short or long breaks throughout whenever the patient feels like it. The objective is to understand, from the perspective of the patient, how they perceive their treatment, what main areas need improving and their general quality of life. The intention through the interviews is not to judge good from bad care directly, but to get an empathetic understanding of the current state of the patient journey. Obtaining the patients’ perspective through interviews is important as they may mention topics, events, or small moments which from the perspective of a care professional may be unimportant or even non‐existent.

In preparation for the interviews, an interview guide is prepared which contains questions to be asked. This is a *guide* and is to be used as such. Empathetic patient interviews are arranged as a guided conversation in which the patient is encouraged to elaborate on topics relevant to them. Subsequently, the interviewer is encouraged to enquire the reasoning behind specific actions, opinions, or moments mentioned by the patient.

The interviewer outlines the project and the reason for the interview as well as informs the patient there are no right or wrong answers, but we are aiming to get their valuable perspective on their treatment.

Interview themes and questions:

- Interviewer will present him/herself and describe shortly about the project.

**Initially its worth outlining the project and interview itself to reassure the patient**:

- This study is sponsored by a pharmaceutical company Takeda
- Your responses will be used by us and the sponsoring pharmaceutical company for patient journey research purposes only
- Your name and personal data will be kept confidential
- Findings from this discussion will be collated with other respondents and presented to the sponsor in aggregated or anonymous form
- Possible adverse events will be reported to Takeda
- You have the right to withdraw from the interview at any time.
- Your answers will be analysed anonymously and summarised. The responses from the interviews are combined and processed in such a way that individual respondents cannot be identified from the summary. The results, including some of your testimonials, may be published to improve the awareness of narcolepsy and the experience of narcolepsy patients.

PATIENT EXPERIENCE

**BACKGROUND**

1. Please introduce yourself and maybe tell us how you like to spend your time.
  - Do you have any hobbies?
  - What kind of work do you or did you do?
  - What kind of things are part of your life?

**PRE‐DIAGNOSIS** (*briefly, not important to go too deep in the first part of the patient journey*)

2 Can you tell me how your journey with narcolepsy started? When did you notice something was different?
3 When you had your first symptoms, did you think it was narcolepsy?
4 What kind of symptoms did you first experience? Please describe them.
  - *Probe*: Did you experience excessive daytime sleepiness (EDS), disrupted night‐time sleep (DNS), hallucinations, sleep paralysis, cataplexy, brain fog?
5 How did you seek help?
  - Did you try to ‘self‐diagnose’ via e.g., internet prior to getting a ‘official’ diagnosis (and were you successful in finding narcolepsy as a potential diagnosis)?
  - Did you try to self‐medicate prior to your official diagnosis? How?
6 When you visited HCP for the first time, were you taken seriously?
7 Did you get any other diagnosis before it was determined narcolepsy or idiopathic hypersomnia?
  - If so, who gave you this diagnosis (GP, specialist etc.)?
8 Have the symptoms changed during the journey? For the better or worse?
9 How many contacts with various healthcare providers prior to getting a diagnosis did you have? Which ones?
10 What was the greatest challenge for you before being diagnosed?

**MOMENT OF DIAGNOSIS**

11 How long did it take from first symptoms until you were diagnosed with narcolepsy?
12 Please describe the diagnosis process, what was it like?
  - Did you have to do specific tests? Which kinds?
  - Do you understand the reason behind specific tests?
  - Do you know if they measured hypocretin levels? (*NOTE: patients in Norway don't want to know*)
  - Overall, was the process clear to you?
  - How did you feel during this process? Why?
13 Who explained to you what narcolepsy is? What was that moment like?
14 Have you tried finding more information about narcolepsy from somewhere?
  - If yes, where?
  - Were you successful? Did you find what you were looking for?
  - Was the information in your local language? Was it understandable?
15 What were your main concerns after hearing about your diagnoses?
16 Were you diagnosed with a specific subtype of Narcolepsy?
  - *If yes*: which type of narcolepsy were you diagnosed with?
  - *If yes*: do you know how they diagnosed you for this specific type?
17 Has your diagnoses changed over time or have you been diagnosed more than once?
  - *If yes*, what have the other NLC/IT diagnoses been before the one you have now?
  - *If yes*, what has being re‐diagnosed or getting another sleep‐diagnosis meant to you?
18 Do you feel you got enough information?
  - How were you told the information? *Probe*: just verbally, given a brochure…
  - Was there information you were hoping to get but you did not?
19 Did you ask questions from the HCPs?
  - Were they able to answer?
  - Do you feel you knew what to ask?
20 Do you feel that HCPs have the right tools or skills for diagnosis or are they missing something?
21 *For patients who were diagnosed as children/teenagers*:
  - When you moved from paediatric care to adult care what was that experience like?
  - Were there any specific positive or negative moments during this transition?

**TREATMENT**

22 What kind of treatment has been offered to you?
  - Are you taking specific medication?
  - Has your doctor prescribed you stimulants and have you received guidance for using them? *If yes*: why are you using the stimulants the way you currently are?
23 Has your ability to function in general been assessed by HCPs? What was that like?
  - *Probe*: how, where, when, by whom? How many times and when?
24 How often do you meet with HCPs? What kind of HCP?
  - What has been the role of GPs in your care?
25 How is your treatment decided upon? Do you feel involved in the decision‐making process?
  - When you are meeting your doctor and they are evaluating the treatment, do they ask questions?
  - Do you fill out questionnaires?
26 What information did you get about your treatment options? Who gave you the information?
  - Would you have preferred to get the information differently? For example, verbally, a brochure, website etc.
  - Looking back, was there any information that could have been helpful to you to have received early on? If yes, which information.
27 Can you describe what the narcolepsy treatment was / is like?
  - Did you stay at hospital/ home?
  - Who took the main responsibility of your treatment?
  - How did you feel during the treatment?
  - *Norway*: are you aware of the new reimbursement system for narcolepsy medications in February 2021? How has it affected your access to medicine?
28 Have you continued with the treatment? Has it helped?
  - Have you stopped treatment / having follow ups? *If yes*, why?
  - Have you chosen not to take any medicine? *If yes*, why?
29 Have you tried various treatment options? Were you informed about any other alternatives than what the HCP could give you?

**LIFE WITH NARCOLEPSY**

30 How many hours do you approximately sleep during the day?
  - Is every day the same?
  - How does this make you feel?
31 What do you consider more important: to able to stay awake as many hours as possible – even if you feel drowsy – or an improvement in your ability to stay alert while you are awake? Why?
32 How many productive hours of daytime do you feel you lose on average in a day/week?
33 Has narcolepsy affected your future plans?
  - Has it affected your job or future ambitions?
  - Has it affected your thoughts about having a family (a partner and/or children)?
34 Do you feel you have been able to maintain your quality of life? Have you been able to live the same way as before?
  - Did the condition stop you from doing something you used to like doing?
  - Do you keep track of your general wellbeing somehow?
  - *If yes*, how? What do you tend to track?
35 Have you had help from a psychologist or other persons regarding your personal wellbeing?
36 Have you tried any methods to improve your daily life? *Such as taking naps, setting routines, taking medication at same time as scheduled naps* etc.
  - Have they worked? Which ones?

**SUPPORT**

37 Are you open about your narcolepsy to other people?
  - How do you feel about the general awareness of narcolepsy? *If low general awareness*: what are things that need to be communicated to people about narcolepsy?
  - How do you feel when explaining narcolepsy to others? What is their reaction?
  - Do you feel there is stigma? *If yes*: what can be done to take away some of the stigma of narcolepsy?
38 Do you feel narcolepsy has affected you in building close relationships with others?
39 Do you know any other patients with narcolepsy? How have you found them?
40 Do you feel the need for more support?
  - *If yes*, what kind of support do you feel you are missing?
41 Did you seek information about narcolepsy from a patient organisation/association?
  - *If yes*, are you active in a patient organisation? Do you participate in the group or simply receive information?
  - *If yes*, how did you find out about the patient organisation?
42 Are you active in groups on social media ‘only’ – without being part of a patient organisation?
43 Do you have ideas of initiatives that can be run by the patient organisation to support you?
44 When the narcolepsy symptoms started, did your parent/caregiver support you and how?
45 Have you received support from family / friends since then? How?
46 Do you have children?
  - *If yes*: do you need and get / have gotten support for their care? What kind of support?
47 What advice would you give to someone who just got diagnosed with narcolepsy?

**DIGITAL SOLUTIONS**

48 Have you ever tried to get into a sleep clinic or hospital for monitoring?
  - *If yes*, what was it like? Did you encounter any hurdles in this? *If yes*, what were they?
49 Are you using any tools for monitoring sleep or symptoms?
  - If yes, what tools do you use?
  - How do use the tools?
  - Do they provide information that your behaviour?
  - Do you share this information with your HCP?
  - What is the value of the digital solutions/ tools for you?
50 Is there something that you feel would be helpful to track, in terms of triggers / symptoms / sleep patterns etc.?
51 Would you use wearables if you feel they provided insights that could help improve your life with narcolepsy?

**CONCLUSION AND CONSENT TO BE CONTACTED**

52 Finally, would it be ok for you, if we reach out again to ask you some more questions or do some further research, if needed?

## APPENDIX C Narcolepsy HCP interview guide.

### C.1

Information for the interviewer:

The following document describes the interview process and contains an overview of the interview questions.

The interviews will take ~60 min. The objective is to understand, from the perspective of the HCP, how patients perceive their treatment, what main areas need improving and their general quality of life. The intention through the interviews is not to judge good from bad care directly, but to get an empathetic understanding of the current state of the patient journey. Obtaining the patients’ perspective through interviews is important as they may mention topics, events, or small moments which from the perspective of a care professional may be unimportant or even non‐existent.

In preparation for the interviews, an interview guide is prepared which contains questions to be asked. This is a *guide* and is to be used as such. Empathetic interviews are arranged as a guided conversation in which the participant is encouraged to elaborate on topics relevant to them. Subsequently, the interviewer is encouraged to enquire the reasoning behind specific actions, opinions, or moments mentioned by the participant.

The interviewer outlines the project and the reason for the interview as well as informs the patient there are no right or wrong answers, but we are aiming to get their valuable perspective on their treatment.

Interview themes and questions:

- Interviewer will present him/herself and describe shortly about the project.

**Initially its worth outlining the project and interview itself to reassure the patient:**

- This study is sponsored by a pharmaceutical company Takeda
- Your responses will be used by us and the sponsoring pharmaceutical company for patient journey research purposes only
- Your name and personal data will be kept confidential
- Findings from this discussion will be collated with other respondents and presented to the sponsor in aggregated or anonymous form
- Possible adverse events will be reported to Takeda
- You have the right to withdraw from the interview at any time.
- Your answers will be analysed anonymously and summarised. The responses from the interviews are combined and processed in such a way that individual respondents cannot be identified from the summary. The results may be published to improve the experience of narcolepsy patients and HCPs, but the results will be anonymous.

HCP EXPERIENCE

**BACKGROUND**

1. Please introduce yourself.
  - What is your current role / position? Do you or your colleagues have special responsibility for narcolepsy in your department? If so, why?
  - Can you estimate roughly how much of your time do you spend on narcolepsy?
  - Can you tell me how your own journey with narcolepsy started?

**PRE‐DIAGNOSIS AND DIAGNOSIS**

2 Pre‐diagnosis: what kind of HCPs do patients with narcolepsy tend to visit?
  - How are they referred to these different HCPs?
  - Is it common that less experienced HCPs from other clinics reach out to you for advice or are there other relevant channels for this support?
3 Pre‐diagnosis: how do patients with narcolepsy tend to differ with regards to symptoms, difficulties in coping, attitude and QoL?
4 Pre‐diagnosis: do you find patients have often been misdiagnosed?
  - *If yes*, which misdiagnosis have they been given? Who diagnosed them?
  - What kind of treatments have they often tried before receiving the correct diagnosis?
5 Pre‐diagnosis: do you sense patients have strong feelings about the healthcare system overall? How do patients discuss about this matter?
  - What is the emotional state of patients once they to a specialist?
  - What is often the level of functionality / ability to work once they are referred to specialists?
6 Norway only: Please describe your co‐operation with the National competence centre (Nevsom)?
7 How do you know to suspect narcolepsy? Are there symptoms which are the clearest signs of narcolepsy? *Probe*: excessive daytime sleepiness (EDS), disrupted nighttime sleep (DNS), hallucinations, sleep paralysis, cataplexy, brain fog
8 When you suspect narcolepsy what do you do?
  - What actions do you take?
  - Do you perform tests? Which ones? What needs to be done to perform these tests?
  - Do you follow any national / local guidelines for narcolepsy diagnosing?
9 Do you diagnose NT1 versus NT2? How?
10 Do you measure hypocretin levels? Why or why not?
11 How do you evaluate functionality and the severity of symptoms? Do you use any standard scales? How do you evaluate the occupational status?
12 What do you tell the patient as you are performing these tests?
13 How is the diagnosis communicated to the patients? What is said or done?
  - *Probe*: is information given to the patients only verbally or is something written down, are they handed a leaflet or shown a website?
14 Is informing patients of the diagnosis difficult?
  - If yes, what could make it easier?
15 Do patients ask you questions when they are informed about their narcolepsy?
  - If yes, what kind of questions? How do you answer?
16 How long does it usually take in your experience from the start of symptoms until receiving the correct diagnosis?
  - Is this length of time a problem / too long?
  - If yes, how could it be improved?
17 Have you had patients who were misdiagnosed or need to be re‐diagnosed?
  - If yes, tell me about them.
18 What would make diagnosing patients easier or faster?
19 What happens immediately after the patients have been informed of the diagnosis?
20 Some patients who are diagnosed as children / teenagers:
  - Is diagnosing children / teenagers different to diagnosing adults? How?
  - When children / teenagers move from paediatric care to adult care, what is that transition like? What is the process?
  - Is there something which needs to be improved in this transition process?

**POST‐DIAGNOSIS AND TREATMENT**

21 How do you decide what kind of treatment is the best for a newly diagnosed patient with narcolepsy?
  - Do you ask additional questions just after diagnosis? Which ones?
  - Who is involved in the treatment decision‐making process? How?
  - Do you conduct additional tests just after diagnosis? Which ones?
  - Do the diagnostic measures/test affect the decision making about the treatment? Does the level of functionality affect the choice?
22 What is the current standard care of narcolepsy in your opinion?
23 What happens next, after patients have been informed of their treatment?
  - How long until they have a follow‐up meeting?
  - Can you describe what a the follow up with patients usually like?
  - Are the follow up meetings usually with the same HCP?
  - *If different HCPs*, does this cause challenges such as different views on diagnosis, treatment, etc?
24 Do you feel patients are adhere to their treatment plans?
  - What may cause them to not comply?
  - What could be improved to enable them to comply easier?
25 Which symptoms are the hardest to treat? Why?
26 In general, what is the role of primary / occupational care in narcolepsy treatment?
  - Are some patients only followed up there?
27 How do you assess if a treatment is working?
  - Do you conduct tests?
  - Do you ask specific questions?
  - Is functionality assessment part of evaluating efficacy of treatment?
28 What causes you to suggest different treatment options to patients?
29 Do you use specific metrics or indicators during diagnosis and treatment? If yes, which ones and how frequently / systematically?
  - What is the outcome of using these metrics or indicators? Does it impact patient care? How?
30 At this stage, how is the functionality and quality of life of patients assessed?
  - Do you use PROMs? Which ones?
  - Do you feel that these metrics and questionnaires are working well?
  - What could be improved or added?
31 Do you have any concerns when prescribing stimulants or other treatment for narcolepsy?
32 Do patients mention finding information about treatments from other sources?
  - If yes, from where? *Prompt*: PAGs, Facebook, books, general internet, etc.
  - Are these sources of information reliable? If no, what are a few good sources of information for patients?
33 Do you offer specific kinds of methods, outside of medical treatment, to improve patients’ lives? *Prompt*: taking naps, setting routines, taking medication at same time as scheduled naps etc.
34 In general, how does narcolepsy tend to affect patients’ quality of life?
  - Do you try to support them in making their QoL better somehow? How?
35 Do you refer patients to sleep clinics?
  - If yes, what are the challenges of referring patient to sleep clinics?
  - Would remote monitoring help? *Probe*: if there were remote EEG monitors, would it help?
36 Do patients tend to use wearables to track their sleep?
  - If yes, do these help them understand their condition better?
  - Are they helpful for you in understanding your patients better?
37 Do patients track their symptoms in general?
  - If yes, what symptoms to patients tend to track?
  - If yes, do they discuss these tracking tools and data in their appointments?
  - Is symptom tracking something which is recommended?

**SUPPORT**

38 Take a moment to consider three very different patients you have helped. Do they mention needing additional (non‐medical) support?
  - How do their needs vary?
  - Do you assist them with these needs? How?
  - Overall, who helps to provide support for patients in need?
39 Are you aware of the patient organisation(s)?
  - What is their role in taking care of patients?
  - Do you recommend patients find support from the patient organisation?
40 Do you involve or take into consideration the patients’ family or caregivers in the treatment plans or care process?
41 From your perspective, what is the role of caregivers in patient care?
  - How do they provide care?
  - How much of the burden of care is on the caregivers?
  - Is there something which could be improved to help caregivers?

**TRANSITION FROM PAEDIATRIC CARE TO ADULT CARE**

42 Do you have experience with patients who have transitioned from paediatric to adult care?
  - *If yes*:
    1. When are younger patients transitioned from paediatric to adult care?
    2. Describe the communication between the responsible HCPs during the transition.
    3. Are there any clear differences in diagnosis / treatment between paediatric / adult care that might affect the patient experience?
    4. Is there a specific HCP assigned to continuously follow‐up a specific patient? Are there differences here with paediatric and adult care?

**CONCLUSION**

43 Do you feel patients with narcolepsy get the best care possible currently?
44 What are the main obstacles you face when providing care for patients with narcolepsy?
  - *Prompt*: patient treatment compliance, Specialist to GP communication, general awareness about narcolepsy, lack of available medication etc
  - What could be improved?
  - How could these be improved?

## APPENDIX D Survey structure.

### D.1

**Basic information**

1. **Which of the following are you currently diagnosed with?**
  - Narcolepsy type 1 (narcolepsy with cataplexy)
  - Narcolepsy type 2 (narcolepsy without cataplexy)
  - Idiopathic hypersomnia
  - I do not know

If NT1 or NT2 or I do not know ‐> answer NT specific questions

If idiopathic hypersomnia ‐> answer IH specific questions

**NARCOLEPSY specific questions** (transfer patient here based on their answer on Q1 but not after they answer Q2)

2 Based on your symptoms, were you originally diagnosed with something other than your current diagnosis?
  - Yes
  - No

If YES, what was your previous diagnosis?

(Add checklist): sleep apnea, IH, NT1, NT2, restless legs syndrome, depression, anxiety, ADHD, insomnia, hormonal disturbance, other: open text

3 **When were you diagnosed with narcolepsy?**
  d dropdown menu: select a year (list 2022‐2005, last option: “before 2005”)
4 How much time did it take from first symptoms until you were diagnosed with narcolepsy? (Select from dropdown)
  - 0–6 months
  - 6–12 months
  - list years to choose from: 1,2,3,4,5…15
  - More than 15 years
5 How long did it take from diagnosis until your treatment significantly improved your quality of life?
  - It has not yet significantly improved
  - 0–3 months
  - 6 months
  - 9 months
  - 1 year
  - 2
  - 3
  - 4
  - 5…15
  - More than 15 years
6 How many hours do you approximately sleep during your usual daytime (between the time you wake up in the morning and the time you go to sleep at night)?
  - Select from dropdown
7 How many hours do you approximately sleep during night? (between the time you go to bed up in the evening and the time you wake up in the morning)?
  - Select from dropdown
8 How many productive hours of daytime do you feel you lose on average in a day due to narcolepsy?
  - Select from dropdown
9 How many days a month are you unable to work/study due to narcolepsy (sick leave or planned absence from work/studies)
  - Select from dropdown
10 How do you keep track of how your narcolepsy is affecting your everyday life? Which of the following options do you use?

Multiple choice (you can choose more than one option):

- Digital tool(s) to track sleep
- Digital tool(s) to track symptoms
- Paper journal
- Electronic journal
- Other (open text)
- I do not actively keep track of my condition

11 Prioritise the Nordic narcolepsy challenges

Based on the Nordic narcolepsy interviews, several challenges with the patient experience were identified. Our goal is to validate, which challenges are most influential to people with narcolepsy.

Please prioritise the following narcolepsy‐related challenges based on your personal experience.

*The challenge you place at the top is the most influential to you. You have maybe experienced this challenge and/or you think that this challenge is causing/could cause problems in your everyday life and for other people with narcolepsy as well*.

*The challenge you place at the bottom is the least important challenge to you. Compared to other challenges, this has not caused any/significant problems in your life*.

*Start by dragging a challenge to the box. If you feel a specific challenge had nothing to do with your narcolepsy experience, you may leave it out of the list. You can then rearrange the order by using the arrows or even remove a challenge from the list by clicking the X symbol. Once you are satisfied with your prioritised list, proceed to the next question*.

**Challenges:**

1 Patients must be proactive to find narcolepsy‐related information
2 Getting a narcolepsy diagnosis was a long and burdensome process
3 Being misdiagnosed at start caused significant consequences (e.g., wrong medication, difficulties during studies, getting/maintaining a job or developing relationships)
4 Lack of information about coping with narcolepsy in daily life
5 Lack of knowledge about narcolepsy among primary care physicians
6 Lack of knowledge about narcolepsy among neurologists responsible for your treatment/follow‐up
7 Lack of psychological support for patients with narcolepsy
8 Finding the suitable medication and treatment is burdensome
9 Lack of support/information about non‐medicinal treatment
10 Follow‐ups with specialists are not regular / provide real value
11 Lack of general awareness about narcolepsy causes social stigma (e.g., being considered lazy)
12 Lack of facilitation in work/school to better cope with narcolepsy challenges in everyday life
12 What kind of improvements, solutions, tools or support services could help you and other people with narcolepsy to address the TOP three challenges you prioritised in the previous question? Please comment on each of the challenges below.
  - Challenge 1:
  - Challenge 2:
  - Challenge 3:
13 Please choose the most suitable option and answer the questions below.

(scale: every day/once a week/once a month/once a year/never)

**During the past year, how often have you…**

- Felt sudden and unarousable sleepiness?
- Felt intolerable sleepiness when sitting, reading or watching television?
- Been feeling sleepy all the time despite sufficient night‐time sleep?
- Had vivid dreams or hallucinations when falling asleep?
- Had difficulty falling asleep during night?
- Experienced sleep paralysis?
- Had difficulty in continuous night‐time sleep, repeatedly waking up and fallen asleep several times?
- Felt fuzzy at wake‐up despite of sufficient night‐time sleep?
- Had difficulty in concentrating and sometimes losing short term memory?
- Lost the strength of a body part when having strong emotions (laughter, anger, fear, excitement, surprise etc) [=cataplexy]?
- Had had drooping or twitching eyelids when emotional (e.g., laughter, anger, fear, excitement, surprise etc.)?
- Been unable to do your job/studies/housework as you would have wished?
- Been unable to stay awake during work/studies/housework?
- Worried about what people think (e.g., ‘I am lazy’)?
- Worried about sudden sleep attacks during important occasions?
- Been unable to keep watching television/movie or enjoying hobbies?
- Been unable to keep up with others in doing tasks
- Been unable to drive a car/bike for a long time
- Gotten tired very easily, no matter what you have been doing
- Needed naps and been unable to work continuously for a long time
- Only been able to give a limited effort at work and experienced a negative impact on job opportunities
- Been worried that narcolepsy could impact your relationship with family / friends?

14 Finally, we would like to know if you have any other medical conditions? You can make multiple selections from the checklist below.
  - Hypercholesterolaemia
  - glucose intolerance
  - hypertension
  - diseases of the digestive system
  - diseases of the heart
  - diseases of the upper respiratory tract
  - diseases of the endocrinopathies
  - Depression
  - Panic disorder
  - PTSD
  - Phobias
  - Social anxiety disorder
  - Insomnia
  - REM sleep behaviour disorder
  - Restless leg syndrome
  - Sleep apnea
  - Non‐REM parasomnias
  - Peripheral neuropathy
  - Headache
  - Chronic lumbar pain
  - Car‐related trauma
  - Other (open text)

**IDIOPATHIC HYPERSOMNIA specific questions (transfer the patient here if they choose ‘IH’ in Q1**

15 Based on your symptoms, were you originally diagnosed with something other than your current diagnosis?
  - Yes
  - No

If YES, what was your previous diagnosis?

(Add checklist): sleep apnea, IH, NT1, NT2, restless legs syndrome, depression, anxiety, ADHD, insomnia, hormonal disturbance, other: open text

16 **When were you diagnosed with idiopathic hypersomnia (IH)?**
  d dropdown menu: select a year (2022‐2005, last option: ‘before 2005’)
17 How much time did it take from first symptoms until you were diagnosed with IH? (Select from dropdown)
  - 0–6 months
  - 6–12 months
  - list years to choose from: 1,2,3,4,5…15
  - More than 15 years
18 How long did it take from diagnosis until your treatment significantly improved your quality of life?
  - It has not yet significantly improved
  - 0–3 months
  - 6 months
  - 9 months
  - 1 year
  - 2
  - 3
  - 4
  - 5…15
  - More than 15 years
19 How many hours do you approximately sleep during your usual daytime (between the time you wake up in the morning and the time you go to sleep at night)?
  - Select from dropdown
20 How many hours do you approximately sleep during night? (between the time you go to bed up in the evening and the time you wake up in the morning)?
  - Select from dropdown
21 How many productive hours of daytime do you feel you lose on average in a day due to IH?
  - Select from dropdown
22 How many days a month are you unable to work/study due to IH (sick leave or planned absence from work/studies)
  - Select from dropdown
23 How do you keep track of how IH is affecting your everyday life? Which of the following options do you use?

Multiple choice (you can choose more than one option):

- Digital tool(s) to track sleep
- Digital tool(s) to track symptoms
- Paper journal
- Electronic journal
- Other (open text)
- I don't actively keep track of my condition

24 Prioritise the Nordic Idiopathic hypersomnia patient challenges

Based on the Nordic narcolepsy interviews, several challenges with the patient experience were identified. Our goal is to validate, which challenges are most influential to people with idiopathic hypersomnia.

Please prioritise the following IH‐related challenges based on your personal experience.

*The challenge you place at the top is the most influential to you. You have maybe experienced this challenge and/or you think that this challenge is causing/could cause problems in your everyday life and for other people with IH as well*.

*The challenge you place at the bottom is the least important challenge to you. Compared to other challenges, this has not caused any/significant problems in your life*.

*Start by dragging a challenge to the box. If you feel a specific challenge had nothing to do with your IH experience, you may leave it out of the list. You can then rearrange the order by using the arrows or even remove a challenge from the list by clicking the X symbol. Once you are satisfied with your prioritised list, proceed to the next question*.

**Challenges:**

1 Patients must be proactive to find IH‐related information
2 Getting an IH diagnosis was a long and burdensome process
3 Being misdiagnosed at start caused significant consequences (e.g., wrong medication, difficulties during studies, getting/maintaining a job or developing relationships)
4 Lack of information about coping with IH in daily life
5 Lack of knowledge about IH among Primary Care physicians
6 Lack of knowledge about IH among neurologists responsible for your treatment/follow‐up
7 Lack of psychological support for patients with IH
8 Finding the suitable medication and treatment is burdensome
9 Lack of support/information about non‐medicinal treatment
10 Follow‐ups with specialists are not regular / provide real value
11 Lack of general awareness about IH causes social stigma (e.g., being considered lazy)
12 Lack of facilitation in work/school to better cope with IH challenges in everyday life
25 What kind of improvements, solutions, tools or support services could help you and other people with IH to address the TOP three challenges that you prioritised in the previous question? Please comment on each of the challenges below.
  - Challenge 1:
  - Challenge 2:
  - Challenge 3:
26 Please choose the most suitable option and answer the questions below.

(scale: every day/once a week/once a month/once a year/never)

**During the past year, how often have you…**

- Felt sudden and unarousable sleepiness?
- Felt intolerable sleepiness when sitting, reading or watching television?
- Been feeling sleepy all the time despite sufficient night‐time sleep?
- Had vivid dreams or hallucinations when falling asleep?
- Had difficulty falling asleep during night?
- Experienced sleep paralysis?
- Had difficulty in continuous night‐time sleep, repeatedly waking up and fallen asleep several times?
- Felt fuzzy at wake‐up despite of sufficient night‐time sleep?
- Had difficulty in concentrating and sometimes losing short term memory?
- Been unable to do your job/studies/housework as you would have wished?
- Been unable to stay awake during work/studies/housework?
- Worried about what people think (e.g., ‘I am lazy’)?
- Worried about sudden sleep attacks during important occasions?
- Been unable to keep watching tv/movie or enjoying hobbies?
- Been unable to keep up with others in doing tasks
- Been unable to drive a car/bike for a long time
- Gotten tired very easily, no matter what you have been doing
- Needed naps and been unable to work continuously for a long time
- Only been able to give a limited effort at work and experienced a negative impact on job opportunities
- Been worried that IH could impact your relationship with family / friends?

27 Finally, we'd like to know if you have any other medical conditions? You can make multiple selections from the checklist below.
  - Hypercholesterolaemia
  - glucose intolerance
  - hypertension
  - diseases of the digestive system
  - diseases of the heart
  - diseases of the upper respiratory tract
  - diseases of the endocrinopathies
  - Depression
  - Panic disorder
  - PTSD
  - Phobias
  - Social anxiety disorder
  - Insomnia
  - REM sleep behaviour disorder
  - Restless leg syndrome
  - Sleep apnea
  - Non‐REM parasomnias
  - Peripheral neuropathy
  - Headache
  - Chronic lumbar pain
  - Car‐related trauma
  - Other (open text)
28 Personal information

Full name:

Email address:

Thank you for sharing your experience! Your answers will inform future initiatives to improve the experience of people living with narcolepsy and idiopathic hypersomnia in the Nordic countries.
